# The role of traditional medicine in providing palliative care and improving the quality of life: a survey on patients’ knowledge and utilization in Kumasi metropolis, Ghana

**DOI:** 10.1186/s12906-025-04961-4

**Published:** 2025-06-09

**Authors:** Antwi Joseph Barimah, Kofi Bobi Barimah, James Dumba, Yaw Boakye Nketiah, Adom-Brobbey David, Tampuri Sheena Mmaa, Asantewaa Nancy Ntow, Owusu Kwadwo

**Affiliations:** 1 College of Health Yamfo, Yamfo, Ghana; 2 Ghana Communication Technology University, Accra, Ghana

**Keywords:** Traditional medicine, Palliative care, Utilization, Kumasi metropolis, Quality of life

## Abstract

**Background:**

According to the World Health Organization (WHO) the utilization of traditional medicine items exceeds that of conventional medications by two to three times on a global scale. Ghana's health system has seen numerous changes and revolutions since gaining political independence, but the nation still struggles to offer its rapidly expanding population an adequate, suitable, complete, and efficient healthcare system. This study assessed the role of traditional medicine in providing palliative care and improving the quality of life while focusing on patients’ knowledge and utilization.

**Methods:**

A hospital-based descriptive cross-sectional study design was employed for the study. The study population consisted of patients who were receiving traditional medical treatment for health conditions such as diabetes and hypertension in the Kumasi metropolitan area. A multi-stage sampling technique was deployed in the study of which 440 participants made up the total sample.

**Results:**

The findings of the study revealed that a substantial proportion of patients (81.04%) demonstrated a strong knowledge of traditional medicine within the context of palliative care. Awareness of traditional medicine was universal among participants (100%), with herbal medicine being the most commonly recognized form (97.39%). Additionally, nearly all participants (99.53%) acknowledged the potential role of traditional medicine in palliative care and affirmed its accessibility (98.82%).

**Conclusion:**

Patients extensively used alternative medicine and showed strong contentment with its success, noting notable enhancements in their health. A lot of people suggest converting traditional medicine into modern dosage forms in order to improve health outcomes and quality of life.

**Supplementary Information:**

The online version contains supplementary material available at 10.1186/s12906-025-04961-4.

## Introduction

Palliative care is a specific sector of the healthcare industry focused on improving the quality of life for people with severe, life-threatening diseases [1]. The main areas of focus are managing pain, relieving symptoms, providing psychological support, and meeting the emotional and spiritual needs of patients [2]. Palliative care is closely connected to cultural beliefs and practices [3]. There has been a growing interest in integrating traditional medicine into the mainstream of healthcare systems to enhance patient well-being and treatment outcomes [4]. The integration of traditional medicine into palliative care represents a promising avenue for providing culturally sensitive and holistic patient-centred care [5].

Despite all the changes and advancements in Ghana's healthcare system since gaining independence, the country continues to face challenges in delivering a quality healthcare system that meets the needs of its expanding population [6]. Preventable transmissible illnesses are still the main reason for sickness and death in Ghana, placing a heavy load on the people [7].

The coexistence of communicable and non-communicable diseases, along with escalating healthcare costs, necessitates extending health services beyond orthodox medicine (OM). This is particularly crucial for addressing challenges posed by HIV/AIDS, malaria, birth complications, and other priority public health issues in Ghana [8]. The numerous health benefits of traditional medicine support its integration into Ghana's healthcare system. Although certain Traditional Medicine (TM) techniques have proven to be helpful for specific conditions, further research is needed to fully understand their benefits [9].

There is a common belief among patients that TM can help with managing pain and relieving symptoms. However, there is a lack of research on the specific impact of TM in palliative care in Ghana [10]. There is a significant lack of understanding about the specific contribution that traditional medicines make in disease treatment in Kumasi, Ghana. Furthermore, individuals in Kumasi possess limited understanding of herbal remedies [11]. This research aimed to fill the gap by exploring the role of traditional medicine in providing palliative care and improving the quality of life. A survey on patients’ knowledge and utilization.

## Methodology

### Study area and design

The research took place in the Kumasi metropolitan area, the main city of the Ashanti Region in Ghana, West Africa, which includes urban and rural areas. Kumasi was selected as the location for the study because of various reasons that made it perfect for assessing the influence of traditional medicine on palliative care and patient's quality of life. The region is abundant in cultural heritage, with longstanding customs of traditional healing handed down through generations, providing a distinctive environment to investigate the blending of traditional and modern medical approaches in palliative care. The urban area has a variety of healthcare facilities, such as advanced hospitals and clinics, as well as traditional healing centres. The research concentrated on Medimafo Herbal Clinic, Dr. Mensah Herbal Clinic, Abbey Herbal Clinic, Gracegift Herbal Clinic, Lawson Herbal Clinic, Amusu Herbal Clinic, all private facilities in Kumasi that offer a wide range of healthcare services. These clinics were chosen because they were easily accessible by road and had a strong transportation infrastructure, making data collection easier. Understanding the differences in the role of traditional medicine across various settings in Kumasi was essential due to the diverse composition of urban and rural communities. The study was conducted between January and June, 2022 within the aforementioned herbal clinics.

### Sampling size determination

The Yamane formula was used for calculating the sample size for the study.

$$\text{n}=\frac{N}{1+N({e)}^{2}}$$

where n is the sample size, N is the population and e is the margin of error.

A 95% CI was used with a 5% margin of error. According to the 2021 Population and Housing Census from the Ghana Statistical Service, the population of the Kumasi Metropolis was 3,490,000. Hence, the sample size was calculated as;

$$\text{n}=\frac{3490000}{1+3490000({0.05)}^{2}}= 399.95,\text{ approximately }400.$$

10% was added to account for non-response rate. A sample size of 440 was therefore used for the study. Stratified sampling was used to select the participants from six (6) herbal clinics within the Kumasi Metropolis.

The estimated number of patients at the six (6) herbal clinics within the Kumasi Metropolis is shown in Table 1 above.

**Table 1:** Estimated population of patients at each herbal clinic

HERBAL CLINIC	ESTIMATED POPULATION
Medimafo Herbal clinic	328
Dr. Mensah Herbal Clinic	208
Abbey Herbal Clinic	189
Gracegift Herbal Clinic	236
Lawson Herbal Clinic	426
Amusu Herbal Clinic	170
**Total**	**1557**

Source: Fieldwork, 2024

The number of respondents needed from the various herbal clinics is displayed in Table 2. 84 respondents were selected from Medimafo Herbal clinic, 53 from Dr. Mensah Herbal Clinic, 49 from Abbey Herbal Clinic, 61 from Gracegift Herbal Clinic, 109 from Lawson Herbal Clinic and 44 from Amusu Herbal Clinic.

**Table 2:** Strata size determination of herbal clinics

HERBAL CLINIC	ESTIMATED POPULATION	SAMPLE FRACTION *n	STRATA SIZE
Medimafo Herbal Clinic	328	328/1557*400	84
Dr. Mensah Herbal Clinic	208	208/1557*400	53
Abbey Herbal Clinic	189	189/1557*400	49
Gracegift Herbal Clinic	236	236/1557*400	61
Lawson Herbal Clinic	426	426/1557*400	109
Amusu Herbal Clinic	170	170/1557*400	44

Source: fieldwork 2024

### Instrument for data collection and procedure

A semi-structured questionnaire was created to collect data from participants regarding different factors pertaining to the objectives of the study. The instrument gathered information on demographic details like age, gender, occupation, and educational level to evaluate their physical, emotional, social, and religious well-being. It also examined traditional medicine's knowledge, usage, efficacy, and satisfaction in palliative care. The instrument consisted of both open-ended and closed inquiries, giving patients the opportunity to select from the responses provided or provide in-depth explanations.

Approval was requested from the Herbal Clinics before starting the data collection process. Following approval, the researchers dedicated a 20-day period towards data collection data to make sure all missing data was documented and followed up on. Respondents permission were also sought before questionnaires were distributed. Questionnaires were given serial numbers, and coded data for variables was duly done. Data cleaning and analysis were conducted with SPSS version 20, which involved biostatistical computations like Pearson chi-square tests. Tables and graphs were made using Microsoft Excel 2016. Descriptive statistics presented data through frequencies and percentages, while bar graphs, bar charts, and pie charts were also depicted. Statistical techniques such as Pearson chi-square tests were employed to analyze relationships between categorical variables, where a *p*-value below 0.05 was deemed statistically significant.

## Results

A total of 440 questionnaires were distributed, however 422 were received, accounting for 95.9% response rate.

The socio-demographic information for respondents were displayed in Table 3.

**Table 3:** Demographic Information of Respondents

Variables	Frequency	Percentages
**Age**		
< 20	2	0.47%
20–29	29	6.87%
30–39	29	6.87%
40–49	89	21.09%
50–59	137	32.46%
60–69	90	21.33%
70–79	38	9.00%
80–89	8	1.90%
**Gender**		
Male	244	57.82%
Female	178	42.18%
**Occupation**		
Farmer	155	36.73%
Health worker	30	7.11%
Teacher	57	13.51%
Trader	27	6.40%
Others	153	36.26%
**Religion**		
Christianity	310	73.46%
Islam	81	19.19%
Traditionalist	30	7.11%
pagan	1	0.24%
**TOTAL**	**422**	**100.00%**

Source: field Survey, 2024

The demographic data provided insights into the demographics of the respondents, indicated the distribution across age, gender, occupation, and religious affiliation.

From Table 3 above, the largest age group was 50–59, comprising 32.46% of respondents, followed by 40–49 (21.09%) and 60–69 (21.33%). The smallest groups were < 20 (0.47%) and 80–89 (1.90%). 57.82% of respondents were male, while 42.18% were female. The most common occupation was Farmer (36.73%), followed by others (36.26%), which included Beautician, Businessman, Businesswoman, Civil Servant, Driver, Electrician, Farmer, and Teacher (13.51%). Health workers and Traders were less common, at 7.11% and 6.40%, respectively. The majority identified as Christian (73.46%), followed by Islam (19.19%) and Traditionalist (7.11%). A small percentage (0.24%) identified as pagan.

### Assessing the level of knowledge of traditional medicine practices among patients in the kumasi metropolitan

Knowledge level of Traditional Practices among Patients in the Kumasi Metropolitan were displayed in Table 4.

**Table 4:** Knowledge and Perception of Traditional Medicine in Palliative Care

Variables	Frequency	Percentages (100%)
**traditional medicine is known by participants**		
Herbal medicine practitioners	411	**97.39%**
Bone setters	3	**0.71%**
Traditional birth attendance	1	**0.24%**
Others	7	**1.66%**
**Source of information**		
Media	276	**65.40%**
Relatives	115	**27.25%**
Friends	30	**7.11%**
Newspapers	1	**0.24%**
**Traditional medicine can be used for palliative care and improve quality of life**		
YES	420	**99.53%**
NO	2	**0.47%**
**adverse effects of traditional medicine**		
No adverse effect	354	**83.89%**
Had adverse effects like skin rash, vomiting, dizziness	57	**13.51%**
Users experienced inexplicable adverse effects	9	**2.13%**
Others ie headaches, heartburn	2	**0.47%**
**Is Health education about the risks and benefits of traditional medicine important?**		
YES	421	**99.76%**
NO	1	**0.24%**
**Integration of traditional medicine**		
YES	400	**94.79%**
NO	22	**5.21%**
**TOTAL**	**422**	**100.00%**

Source: survey field, 2024

From Table 4, out of the 422 participants, herbal medicine practitioners were widely known, with 97.39% indicating awareness. Bone setters and traditional birth attendants were less known, mentioned by only 0.71% and 0.24% of participants, respectively. Additionally, 1.66% of participants mentioned other forms of traditional medicine. The majority (65.40%) heard about traditional medicine through media sources, while relatives contributed to awareness for 27.25% of participants.

A vast majority (99.53%) of participants were aware that traditional medicine could be used for palliative care and improving quality of life. 83.89% of participants believed traditional medicine had no adverse effects, while 13.51% experienced adverse effects like skin rash, vomiting, or dizziness. 2.13% reported inexplicable adverse effects, and 0.47% mentioned other adverse effects like headaches or heartburn. Almost all participants (99.76%) agreed that health education about the risks and benefits of traditional medicine was important.

Regarding the perception of traditional medicine in palliative care, a significant majority (94.79%) believed that traditional medicine could complement modern healthcare in providing palliative care. Only a very small percentage (0.47%) disagreed with this notion.

### Knowledge score of respondents on traditional medicine in palliative care

To evaluate participants'knowledge of traditional medicine in palliative care, points were assigned based on their responses to several questions. Participants received 1 point for having heard of traditional medicine (TM), 1 point for each type of TM known (e.g., herbal medicine), 1 point for being aware that TM could be used in palliative care, 1 point for each correct response about the adverse effects of TM, 1 point for recognizing the importance of health education about TM, and 1 point for perceiving that TM could complement modern healthcare. The total possible score for each participant was 6 points.

The knowledge scores were categorized into three groups: Poor (0–2), Moderate (3–4), and Good (5–6). The frequency of participants in each category was then counted. To provide a clear understanding of the distribution, percentages were calculated by dividing the frequency of each category by the total number of participants (422) and multiplying by 100.

Table 5 showed that a significant majority (81.04%) of participants had good knowledge of traditional medicine in palliative care, while 18.96% had a moderate understanding. There were no participants with poor knowledge, indicating a generally well-informed group.

**Table 5:** rating of knowledge of respondents on traditional medicine in palliative care

KNOWLEDGE SCORE	SCALE	FREQUENCY	PERCENTAGES
POOR	0–2	0	0.00%
MODERATE	03–04	80	18.96%
GOOD	05–06	342	81.04%
TOTAL		422	100.00%

Source: survey field, 2024

### Utilization and practice of traditional medicine in palliative care

Out of the 422 participants, Table 6 above indicated a vast majority (98.82%) of respondents believed that traditional medicine was accessible. Herbal medicine was the most commonly used type, with 99.29% of respondents indicating its usage. Bone setters were used by a very small percentage (0.71%) of respondents. The most common diseases treated with traditional medicine were hypertension (29.15%) and diabetes (26.78%). Other diseases treated included hepatitis B (21.56%), stroke (15.17%), and various others such as liver cirrhosis, hernia, candidiasis, stomach ulcer, kidney disease, general pains, and loss of appetite, malaria, fibroid, and fatty liver (7.35%).

**Table 6:** Utilization and Practice of Traditional Medicine in Palliative Care

Variables	Frequency	Percentages
**Do you believe traditional medicine is accessible?**		
YES	417	**98.82%**
NO	5	**1.18%**
**Type of traditional medicine used for managing condition**		
Herbal medicine	419	**99.29%**
Bone setters	3	**0.71%**
Traditional birth attendance	0	**0.00%**
**What disease did you use traditional medicine to treat?**		
Hypertension	123	**29.15%**
Diabetes	113	**26.78%**
Stroke	64	**15.17%**
Hepatitis B	91	**21.56%**
Others ie, Liver cirrhosis, Hernia, candidiasis, and Stomach ulcer. Kidney disease, General Pains and Loss of appetite, malaria, fibroid, fatty liver	31	**7.35%**
**Recommend the use of Traditional Medicine in the community**		
YES	422	**100.00%**
NO	0	**0.00%**
**Used of modern medicine**		
YES	413	**97.87%**
NO	9	**2.13%**
**Traditional Medicines is better than Modern Medicine**		
YES	408	**98.79%**
NO	5	**1.21%**
**TOTAL**	**413**	**100.00%**
**Integration of Traditional Medicine with Modern Medicine to improve healthcare coverage**		
YES	414	**98.10%**
NO	8	**1.90%**
**TOTAL**	**422**	**100.00%**

Source: survey field, 2024

All respondents (100.00%) recommended the use of traditional medicine in the community. The majority (97.87%) of respondents had also used modern medicine. A high percentage (98.79%) of respondents believed that traditional medicine was better than modern medicine. A significant majority (98.10%) of respondents supported the integration of traditional medicine with modern medicine to improve healthcare coverage.

### The effectiveness and satisfaction of traditional medicine among patients

Table 7 above indicates that most patients (96.68%) found traditional medicine effective in addressing their health concerns. Only a small percentage (1.18%) found it ineffective, and 2.13% had a neutral opinion. Traditional medicine was reported to be extremely helpful in managing signs/symptoms by 81.75% of patients, moderately helpful by 18.01%, and only 0.24% reported it as not helpful. 79.62% of patients did not experience any side effects or adverse reactions from using traditional medicine, 12.32% reported experiencing side effects, and 8.06% were unsure.

**Table 7:** The effectiveness and satisfaction of traditional medicine among patients

Variables	Frequency	Percentages
**The effectiveness of traditional medicine**		
**Effectiveness of traditional medicine in addressing their health concerns**		
Ineffective	5	**1.18%**
Neutral	9	**2.13%**
Effective	408	**96.68%**
**Extent traditional medicine helped in managing signs/symptoms**		
Not at all	1	**0.24%**
Moderately	76	**18.01%**
Extremely	345	**81.75%**
**Side effects or adverse reactions from using traditional medicine**		
Yes	52	**12.32%**
No	336	**79.62%**
Don’t know	34	**8.06%**
**Improvement in your overall well-being after using traditional medicine**		
No improvement	2	**0.47%**
Moderate Improvement	141	**33.41%**
Significant Improvement	279	**66.11%**
**Satisfaction with Traditional Medicine**		
**How satisfied are you with the use of traditional medicine treatments?**		
Dissatisfied	7	**1.66%**
Neutral	14	**3.32%**
Satisfied	401	**95.02%**
**Would you recommend traditional medicine to others based on your satisfaction?**		
YES	414	**98.10%**
NO	8	**1.90%**
**TOTAL**	**422**	**100.00%**

Source: survey field, 2024

A significant majority (66.11%) of patients reported experiencing significant improvement in their overall well-being after using traditional medicine. 33.41% reported moderate improvement, while only 0.47% reported no improvement. The majority of patients (95.02%) were satisfied with the use of traditional medicine treatments. Only a small percentage (1.66%) were dissatisfied, and 3.32% had a neutral opinion. Almost all patients (98.10%) would recommend traditional medicine to others based on their satisfaction.

### Chi- square analysis test on effectiveness, with some independent variables on traditional medicine

From Table 8 above, the chi-square test yielded insightful findings on the associations between the effectiveness of traditional medicine and various independent variables. Notably,the results, presented in Table 8, revealed that modernizing TM dosage forms (*p*-value: 0.004) and believing in its ability to treat conditions beyond conventional medicine (*p*-value: 0.020) were strongly associated with positive perceptions of effectiveness. Furthermore, effective symptom management (*p*-value: 0.000), improvement in overall well-being (*p*-value: 0.000), and clear communication about treatment (*p*-value: 0.000) were also significantly linked to higher perceived effectiveness. Conversely, experiencing side effects (*p*-value: 0.019) was associated with reduced perceived effectiveness. Interestingly, factors such as age (*p*-value: 0.829), gender (*p*-value: 0.944), occupation (*p*-value: 0.726), and religion (*p*-value: 0.726) did not seem to significantly influence how people perceived the effectiveness of TM. This research suggests that modernization, belief in its capabilities, effective symptom management, clear information provision, and minimizing side effects are key factors in enhancing the perceived effectiveness of traditional medicine.

**Table 8:** Chi-square analysis test on effectiveness of traditional medicine with some independent variables

Attribute	Effective (%)	Ineffective(%)	Total (%)	*p*-value
**Age**				
< 20	0	0.47	0.47	0.829
20–29	6.64	0.24	6.87	
30–39	6.16	0.71	6.87	
40–49	20.62	0.47	21.09	
50–59	32.94	0.95	33.89	
60–69	19.43	0.47	19.91	
70 +	17.10	0.67	17.77	
**Gender**				
Male	40.52	1.42	41.94	0.944
Female	56.16	1.90	58.06	
**Occupation**				
Farmer	35.78	0.95	36.73	0.726
Health worker	7.11	0.00	7.11	
Teacher	33.18	1.66	34.83	
Trader	13.51	0.24	13.74	
Others	7.11	0.47	7.58	
**Religion**				
Christianity	71.09	2.13	73.22	0.726
Islam	18.48	0.71	19.19	
Pagan	0.47	0.00	0.47	
Traditionalist	6.64	0.47	7.11	
**Formulating TM in a modern dosage form will be good enough to treat disease**				
Yes	96.20	3.09	99.29	**0.004**
No	0.48	0.24	0.71	
**Believe that Traditional Medicines can cure some diseases that cannot be treated by Modern Medicine**				
Yes	93.84	2.84	96.68	**0.020**
No	2.84	0.47	3.32	
**Extent at which traditional medicine help in managing symptoms**				
Extremely	80.57	0.95	81.52	**0.000**
Moderately	15.88	2.13	18.01	
Not at all	0.24	0.24	0.48	
**Experiencing any side effects or adverse reactions from using traditional medicine**				
Yes	11.37	0.95	12.32	**0.019**
No	77.96	1.66	79.62	
I don’t know	7.35	0.71	8.06	
**Rate of improvement in overall well-being after using traditional medicine**				
Significant Improvement	65.40	0.95	66.35	**0.000**
Moderate Improvement	31.04	2.13	33.18	
No Improvement	0.24	0.24	0.48	
**Satisfaction with information provided by traditional medicine practitioners**				
Satisfied	92.42	2.37	94.79	**0.000**
Neutral	2.61	0.71	3.12	
Dissatisfied	1.66	0.24	1.90	

Source: survey field, 2024

## Discussion

This study showed that all 422 participants were knowledgeable about traditional medicine, which is significantly more than in previous studies in Jimma town (99.3%) and Jara town (96.3%) [12]. Herbal medicine was the most acknowledged form, with 97.39% recognizing its practice, in line with previous studies [13].

When it comes to where they get their information from, most people (65.40%) rely on media as their main source, with relatives being a distant second (27.25%). Friends made up 7.11% while newspapers made up 0.24%. In Southwest Ethiopia, a study found that family and friends were more important in sharing information compared to the aforementioned situation.

In general, 81.04% of the participants demonstrated satisfactory understanding of traditional medicine in palliative care, consistent with earlier studies [13]. The high level of knowledge in Debre Tabor Town (80.1%) exceeded rates found in Shopa Bultum (69.53%) [14], showing widespread acknowledgment of the benefits of traditional medicine and its importance in healthcare.

According to the study, herbal medicine was the most frequently utilized type, with 99.29% of participants indicating its use, exceeding the rates observed in studies from other nations. In underdeveloped countries, 80% of people depend on traditional remedies for curing different illnesses [15]. A study conducted by Agize, Asfaw, Nemomissa, and Gebre [16] found that patients primarily rely on herbal remedies sourced from natural forests, with leaves being the most commonly utilized. These results align with research conducted in North Central, West, and Southeast Ethiopia [13]. In a study by Chali, Hasho, and Koricha, it was found that a higher percentage, 54.4%, of participants preferred using medical herbs as traditional medicine. In contrast, in a different study conducted in Jimma Town, over half of the participants did not favor traditional medicine [17].

In this research, the primary illnesses managed with traditional medicine were high blood pressure (29.15%) and sugar diabetes (26.78%). Additional illnesses encompassed hepatitis B (21.56%), stroke (15.17%), and different ailments like liver cirrhosis, hernia, candidiasis, stomach ulcer, kidney disease, general pains, loss of appetite, malaria, fibroid, and fatty liver (7.35%). Aragaw, Afework, and Getahun [13] also found that 35.8% of the population utilized traditional medicine for various health issues such as abdominal cramps, abortion, burns, chest pain, cough, cutaneous leishmaniasis, diarrhea, dry cough, fungal infections, gastritis, goitre, hemorrhoids, herpes zoster, hypertension, impotence, infected wounds, joint pain, liver disease, malaria, among other conditions. Nevertheless, the findings contrast with a survey conducted in Shopa Bultum, Southeast Ethiopia, where 72.85% of participants sought help from traditional healers [14]. Cultural acceptability, respect for healers, and accessibility of traditional medicine compared to modern medicine could explain the differences, while similarities might show an evolution of curative practices following disease patterns.

A large majority of participants (98.79%) prefer traditional medicine over modern medicine, surpassing the 79.47% of healthcare providers in Shopa Bultum who support traditional medicine practitioners delivering crucial healthcare services [14]. In contrast to a study by Chali, Hasho, and Koricha, where just 9.2% favored traditional medicine, this discovery suggests modern medicine's promotion and availability in the community outweigh cultural acceptance and perceived effectiveness of traditional medicine [17].

The findings also indicated that a large majority (98.10%) of participants endorse blending traditional and modern medicine to enhance healthcare, in line with a survey in the Shirka district where 84% of modern healthcare providers backed integration. A research conducted in Ghana highlighted the importance of traditional and modern medicine practitioners working together to improve healthcare services, despite facing obstacles such as discrimination, limited understanding, and a shortage of equipment [18]. Yet, additional research indicates that these partnerships can be complicated and frequently lack efficiency.

According to this study, almost all patients (96.68%) reported that traditional medicine was successful in treating their health issues. It was noted that traditional medicine was highly effective in alleviating symptoms in 81.75% of patients. A large majority (66.11%) experienced better overall well-being by using traditional medicine, consistent with Choi’s [13] discovery that Chinese patients found relief from lower back pain, nausea, and knee osteoarthritis with herbal medicine. The use of traditional medicine led to favorable results, including quicker disappearance of clinical symptoms, faster recovery, shorter hospital stays, improved CT images, and higher rates of clinical cure [19].

According to Kaur et al. [20], the satisfaction rate of patients with traditional medicine treatments was high at 95.02%, similar to the 99.4% satisfaction rate among patients in Malaysia for traditional and complementary medicine (T&CM) services, with 91.8% reporting positive health impacts. This percentage is greater than the satisfaction rate reported by Ahmadi et al. [21] in Ethiopia, where 66–80% of patients were content with complementary medicine. In the same way, Shang et al. [22] discovered that 75.5% of individuals in China expressed contentment with traditional Chinese medicine (TCM), with no notable distinctions between TCM and non-TCM medical facilities. Chali, Hasho, and Koricha [17] observed that numerous patients believe traditional medicine can alleviate symptoms even if it does not treat the root cause. Critics in Western settings may discredit traditional medicine, but it continues to be widely embraced in developing nations where a large number of people depend on it for maintaining their health [23]. Surveys on patient satisfaction have been highly important in improving quality and evaluating the success of conventional medical treatments [24].

In this research, 79.62% of individuals did not encounter any side effects or negative responses from conventional medicine, unlike the findings of Aragaw, Afework, and Getahun [13], where 28.3% of subjects had minor side effects. The difference may result from variations in sample sizes, availability of healthcare facilities, or the quality of healthcare services, leading to reduced rates of adverse reactions in our research.

Demographic factors like age, gender, occupation, and religion had *p*-values greater than 0.05 and were not significant in relation to traditional medicine efficacy or satisfaction, in contrast to previous research. Xia and colleagues discovered that age, gender, level of education, and location of residence impacted the effectiveness of traditional medicine. Wang and Xia [25] also emphasized the importance of age, gender, and location in the development of unbalanced constitutions, but there has been no comprehensive national survey to examine how demographic factors impact traditional medicine results. Nevertheless, our research identified noticeable variations (p < 0.001) in perceived efficacy and contentment among participants regarding contemporary forms of traditional medicine, disease control, alleviation of symptoms, and enhancement of general well-being. This indicates that personal beliefs and personal experiences have a greater impact on the efficacy and satisfaction of traditional medicine compared to demographic characteristics.

## Conclusion

The study in the Kumasi Metropolis underscores the importance of traditional medicine in enhancing the quality of life and providing palliative care for patients. The results indicate a strong awareness and favorable view of its efficacy in treating different health issues. There was also a lot of backing for health education to encourage the safe usage of traditional remedies.

Patients extensively used alternative medicine and showed strong contentment with its success, noting notable enhancements in their health. A lot of people suggest converting traditional medicine into modern dosage forms in order to improve health outcomes and quality of life.

The research highlights the importance of acknowledging traditional medicine as a valuable addition to modern healthcare. The popular faith in its effectiveness and patients'readiness to suggest it underscore its lasting significance. Efforts must persist in merging traditional and modern healthcare systems to guarantee all-encompassing healthcare coverage.

## Supplementary Information

Supplementary Material 1

## Data Availability

Data is provided within the manuscript.

## Abbreviations

WHO: World Health Organization
GHS-ERC: Ghana Health Service Ethics Review Committee
OM: Orthodox medicine
TM: Traditional Medicine
HIV/AIDS: Human immuno-deficiency virus; Acquired immune deficiency syndrome
