# Supplementary material for: The role of traditional medicine in providing palliative care and improving the quality of life: a survey on patients’ knowledge and utilization in Kumasi metropolis, Ghana
Source: BMC Complement Med Ther. 2025 Jun 9;25:212. doi: 10.1186/s12906-025-04961-4 (PMC12147286; doi:10.1186/s12906-025-04961-4)
Supplement: Supplementary file 1 — Supplementary Material 1 [file 12906_2025_4961_MOESM1_ESM.docx]

# Appendix: Questionnaire

Dear respondent,

We are researchers from College of Health Yamfo, researching into **‘‘the Role of Traditional Medicine in Providing Palliative Care and Improving Quality of Life: A Survey on Patient's Knowledge and Utilization**”**.** The purpose of the study and its findings is purely academic. We would be grateful if you could devote some of your quality time to studying and answering this questionnaire. All answers will be treated as confidential and will be used for statistical analysis and research purposes only.

If you agree to participate in the study, kindly sign in the column below

Signature……………………………………………..

Thank you.

*Please complete the following by ticking [√] the appropriate answers or writing a brief information in the space provided where necessary*

Section A: Demographic Information of respondents

| A1 | What is your age? | ……………………………………….YEARS | |
| --- | --- | --- | --- |
| A2 | What is your gender? | Male  Female | [ ]  [ ] |
|  | What is your Occupation? | Health worker  Teacher  Farmer  Others | [ ]  [ ]  [ ]  ………………………. |
|  | What is your religious background? | Christianity  Islam  Traditionalist  Others | [ ]  [ ]  [ ]  ……………………….. |

Section B: Knowledge and Perception of Traditional Medicine in Palliative Care

| B1 | Have you ever heard about TM? | Yes  No | [ ]  [ ] |
| --- | --- | --- | --- |
| B2 | Which one do you know? | Herbal medicine  Bone setters  Traditional birth attendance  Others/specify | [ ]  [ ]  [ ]  ………………………………… |
| B3 | Have you visited modern healthcare services soon after visiting a traditional medicine practitioner? | Yes  No | [ ]  [ ] |
| B4 | IF YES, Why did he or she do it? | No improvement  Peer influence  Side effect  Other | [ ]  [ ]  [ ]  ………………………………… |
| B5 | What were the main sources of herbal products? | From practitioners  Relatives  Neighbours  Friends  Themselves | [ ]  [ ]  [ ]  [ ]  [ ] |
| B6 | What do you know about the adverse effects of TM | No adverse effect  Had adverse effects like skin rash, vomiting, dizziness  Users experienced inexplicable adverse effects  Others | [ ]  [ ]  [ ]  ………………………………… |
| B7 | Health education about the risks and benefits of traditional medicine is important. | Yes  No | [ ]  [ ] |
| B8 | Traditional medicines are more effective and safer than modern health services | Yes  No | [ ]  [ ] |

Section c: Utilization and practice of Traditional Medicine in Palliative Care

| C1 | Do you use traditional medicine? | Yes  No | [ ]  [ ] |
| --- | --- | --- | --- |
| C2 | If YES, which of them? | ………………………………………………………………………………………………………………………………………………………………………….. | |
| C3 | Why did you use herbal medicine? | Fever  Malaria  Hypertension  Diabetes  Infection  Constipation  Others/specify | [ ]  [ ]  [ ]  [ ]  [ ]  [ ]  ………………………………… |
| C4 | Do you recommend the use of TM in the community? | Yes  No | [ ]  [ ] |
| C5 | Do you believe that TMs are still accepted and available at an affordable cost in the community? | Yes  No | [ ]  [ ] |
| C6 | Do you believe that breaking the secrecy of TMs may lead to a loss of effectiveness? | Yes  No | [ ]  [ ] |
| C7 | Do you support the integration of MM with TM to improve healthcare coverage? | Yes  No | [ ]  [ ] |
| C8 | Do you believe that TMs can cure some diseases that cannot be treated by MM? | Yes  No | [ ]  [ ] |
| C9 | Do you believe that TMs can cure some diseases that cannot be treated by MM? | Yes  No | [ ]  [ ] |
| C10 | Do you think that if TMs are formulated in a modern dosage form, it will be good enough to treat diseases with an appropriate dose and route? | Yes  No | [ ]  [ ] |

**SECTION D: The effectiveness and satisfaction of traditional medicine among patients**

| The effectiveness of traditional medicine | | | |
| --- | --- | --- | --- |
| D1 | How would you rate the overall effectiveness of traditional medicine in addressing your health concerns? | Very Ineffective  Ineffective  Neutral  Effective  Very Effective | [ ]  [ ]  [ ]  [ ]  [ ] |
| D2 | To what extent has traditional medicine helped in managing your symptoms? | Not at all  Slightly  Moderately  Very  Extremely | [ ]  [ ]  [ ]  [ ]  [ ] |
| D3 | Have you experienced any side effects or adverse reactions from using traditional medicine? | Yes  No  Don’t know | [ ]  [ ]  [ ] |
| D4 | How would you rate the improvement in your overall well-being after using traditional medicine? | No improvement  Moderate Improvement  Significant Improvement | [ ]  [ ]  [ ] |
| **Satisfaction with Traditional Medicine** | | | |
| D5 | How satisfied are you with the information traditional medicine practitioners have about the treatments? | Very dissatisfied  Dissatisfied  Neutral  Satisfied  Very Satisfied | [ ]  [ ]  [ ]  [ ]  [ ] |
| D6 | Would you recommend traditional medicine to others based on your experience? | Definitely Not  Probably Not  Neutral  Probably Yes  Definitely Yes | [ ]  [ ]  [ ]  [ ]  [ ] |
